# Clinical Manifestations of Senecavirus A Infection in Neonatal Pigs, Brazil, 2015

**DOI:** 10.3201/eid2207.151583

**Published:** 2016-07

**Authors:** Raquel A. Leme, Thalita E.S. Oliveira, Brígida K. Alcântara, Selwyn A. Headley, Alice F. Alfieri, Ming Yang, Amauri A. Alfieri

**Affiliations:** Universidade Estadual de Londrina, Paraná, Brazil (R.A. Leme, T.E.S. Oliveira, B.K. Alcântara, S.A. Headley, A.F. Alfieri, A.A. Alfieri);; National Centre for Foreign Animal Disease, Winnipeg, Manitoba, Canada (M. Yang)

**Keywords:** Seneca Valley virus, picornavirus infection, swine, pathogenesis, viruses, piglets, Brazil, Senecavirus A

## Abstract

We identified new clinical manifestations associated with Senecavirus A infection in neonatal piglets in Brazil in 2015. Immunohistochemical and molecular findings confirmed the association of Senecavirus A with these unusual clinical signs and more deaths. Other possible disease agents investigated were not associated with these illnesses.

Senecavirus A (SVA), formerly called Seneca Valley virus, is the single representative species of the genus *Senecavirus* (family *Picornaviridae*). SVA is a single-stranded, positive-sense, nonenveloped RNA virus with a genome size of ≈7.2 kb (*1*).

SVA infection was associated with porcine idiopathic vesicular disease (PIVD) in pigs in Canada (*2*), the United States (*1*), and Brazil (*3*,*4*). The clinical manifestations of PIVD are indistinguishable from those of other vesicular virus infections, including foot-and-mouth disease virus (FMDV), vesicular stomatitis virus, swine vesicular disease virus (SVDV), and vesicular exanthema of swine virus (*2**,**3*). These clinical signs include fluid-filled and ruptured vesicles and ulcerative lesions at the coronary band, hooves, and/or snout (*1*–*4*). In 2015, we identified new clinical manifestations associated with SVA infections in piglets in Brazil.

## The Study

Since early 2015, increased numbers of deaths were recorded in pig herds from different geographic regions of Brazil. Piglets during their first week of life demonstrated clinical signs such as muscular weakness, lethargy, excessive salivation, cutaneous hyperemia, neurologic manifestations, and diarrhea; some died suddenly. Clinical signs lasted for 3–10 days and then disappeared in piglets that survived.

To determine the cause of these illnesses, we investigated 5 farms (A–E). Pig populations per farm varied from 10,000 to 23,000 animals, and piglet death rates during the first week of life ranged from 20% to 30%. Ten piglets that died spontaneously were examined (Table 1).

**Table 1:** Geographic locations and other charactereristics of pig farms affected by Senecavirus A, Brazil, 2015

Farm	State/region	Month of collection	Animal no.	Age, d	Principal clinical manifestations
A	Paraná/Southern Brazil	February	1	2	Weakness at birth, sudden death at 1–3 d of age
			2	1	
B	Paraná/Southern Brazil	February	3	2	Weakness at birth, sudden death at 1–3 d of age
			4	1	
C	Mato Grosso do Sul/Midwest Brazil	March	5	3	Cutaneous hyperemia, diarrhea, excessive salivation, lethargy, death
D	Santa Catarina/Southern Brazil	March	6	2	Acute diarrhea and/or wasting, death
E	Santa Catarina/Southern Brazil	July	7	2	Diarrhea, neurologic manifestations, sudden death
			8	2	
			9	4	
			10	5	

Farms A, B, D, and E had gestating or farrowing sows with fluid-filled and/or ruptured vesicles at the coronary bands, hooves, or snouts; reproductive disorders were not observed. We had identified SVA RNA from sows at farms A and B (*3*) a week before the onset of clinical manifestations in these piglets.

Routine necropsies of all piglets were conducted soon after death. Tissues were fixed by immersion in 10% buffered formalin solution and processed for histopathologic evaluation. Selected tissue fragments were used in an immunohistochemical (IHC) assay designed with monoclonal antibodies to detect SVA (*5*). Duplicate sections of the organs and scrapings from oral vesicles and cutaneous lesions were collected for molecular diagnostics. From piglets at farms C, D, and E, we collected diarrheic fecal samples to investigate the possibility of enteric viruses. We analyzed 81 tissue samples and 6 diarrheic fecal samples during this study by a combination of pathologic and molecular diagnostic methods.

Molecular assays were conducted to identify viruses that might be associated with the reported clinical signs; these included SVA (*3*); FMDV, vesicular stomatitis virus, and SVDV (*6*); teschovirus A, sapelovirus A, and enterovirus G (*7*); porcine parvovirus (*8*); and porcine circovirus type 2 (*9*). Feces and fragments of the small intestine from piglets of farms C, D, and E were evaluated for porcine rotavirus species A, B, C (*10*), and H (*11*); porcine epidemic diarrhea virus (*12*); swine deltacoronavirus (*13*); and transmissible gastroenteritis virus (*12*).

Seventeen amplified products were submitted for sequencing. We conducted sequence identity matrix using BioEdit software version 7.1.11 (http://www.mbio.ncsu.edu/bioedit/bioedit.html). A phylogenetic tree based on nucleotide sequences was obtained using MEGA6 software (http://www.megasoftware.net).

**Figure 1:**
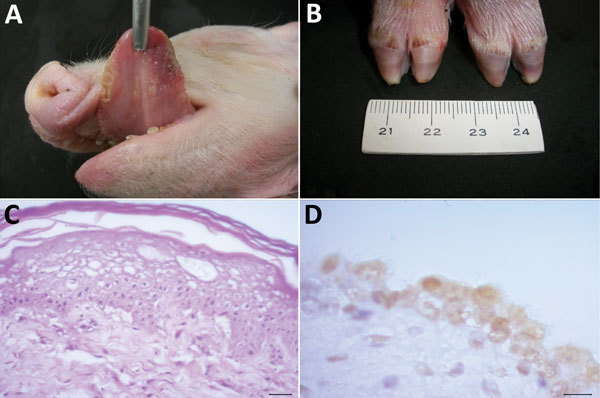
Pathologic alterations in piglets infected with Senecavirus A, Brazil, 2015. Gross examination shows multifocal diphtheric glossitis (A) and ulcerations of the coronary band (B). Histopathologic images demonstrate ballooning degeneration of the epithelium of the tongue (C) and positive immunoreactivity of the uroepithelium of the urinary bladder (D) to Senecavirus A. Panel B, scale shown in centimeters; panel C, hematoxylin and eosin stain; scale bar indicates 20 μm; panel D, immunoperoxidase; scale bar indicates 10 μm.

The most frequent gross manifestations observed were petechial hemorrhages of the kidney (7 piglets) and ulcerative lesions at the tongue (6 piglets) and coronary bands (4 piglets) (Figure 1, panels A, B). Interstitial pneumonia, the predominant histopathologic alteration, occurred in all the piglets; other frequent lesions were diphtheric glossitis (6 piglets), lymphocytic myocarditis (6 piglets), ballooning degeneration of the transitional epithelium of the urinary bladder (Figure 1, panel C) and the ureters (4 piglets), and lymphoplasmacytic encephalitis (3 piglets).

Consistent SVA IHC staining occurred at the transitional epithelium of the renal pelvis and the urinary bladder (Figure 1, panel D) of 4 piglets; within epithelial cells of the choroid plexus of the cerebrum (8 piglets) and the tongue (5 piglets); and at the ependymal cells of the choroid plexus, vascular endothelium, and the enterocytes of the villi of the small intestine (2 piglets) (Table 2).

**Table 2:** Distribution of nucleic acid and antigens of Senecavirus A in 1–5-day-old piglets, Brazil, 2015*

Farm, piglet no.	Identification of Senecavirus A		
	Organs	RT-PCR	IHC
A			
1	Tongue	+	+
	Gingiva	+†	+
	Ruptured cutaneous vesicle (thorax)	+	–
	Coronary band ulcerations	+	ND
	Myocardium	+†	–
	Lung	+	–
	Liver	–	–
	Renal pelvis	+†	+
	Cerebrum	–	+§
	Cerebellum	+	–
	Brainstem	–	–
2	Tongue	+	+
	Myocardium	+†,‡	–
	Lung	+	–
	Liver	+	–
	Renal pelvis	+†	+
	Cerebrum	–	+§
	Cerebellum	+	–
	Brainstem	+	–
B			
3	Tongue	+	+
	Coronary band ulceration	+	ND
	Myocardium	+†	–
	Lung	+	–
	Liver	+	–
	Renal pelvis	+†	+
	Brain	–	+
	Cerebellum	+	–
	Brainstem	+	–
4	Tongue	+	+
	Gingiva	+†	+
	Myocardium	+†	–
	Lung	+	–
	Liver	+	–
	Renal pelvis	+†,‡	+
	Cerebrum	+	+§
	Cerebellum	+	–
	Brainstem	+†	–
C, 5	Myocardium	+	–
	Lung	+†,‡	–
	Liver	–	–
	Small intestine with fecal content	+	+
D, 6	Small intestine with fecal content	+†,‡	+
E			
7	Myocardium	–	–
	Lung	–	–
	Spleen	+†	–
	Renal pelvis	–	+
	Urinary bladder	+	+
	Small intestine with fecal content	–	–
	Cerebrum	–	+§
	Cerebellum	–	–
	Brainstem	–	–
8	Myocardium	+	–
	Lung	+†	–
	Spleen	+	–
	Renal pelvis	+	+
	Urinary bladder	+	+
	Small intestine with fecal content	–	–
	Cerebrum	+	+§
	Cerebellum	+	–
	Brainstem	–	–
9	Gingiva	+	+
	Myocardium	+	–
	Lung	+	–
	Spleen	+	–
	Renal pelvis	+	+
	Urinary bladder	+†	+
	Small intestine with fecal content	+	–
	Cerebrum	–	+§
	Cerebellum	–	–
	Brainstem	+	–
10	Tongue	+†,‡	+
	Gingiva	+	+
	Myocardium	+	–
	Lung	+	–
	Spleen	+	–
	Renal pelvis	+	+
	Urinary bladder	+	+
	Small intestine with fecal content	+	–
	Cerebrum	+	+§
	Cerebellum	+	–
	Brainstem	–	–

*IHC, immunohistochemistry; ND, not done; RT-PCR, reverse transcription PCR; +, positive; –, negative. †Amplicons submitted for sequence analysis. ‡Amplicons representative of each farm in the phylogenetic tree construction. §Choroid plexus.

The expected SVA RNA fragment was amplified by reverse transcription PCR from 77.8% (63/81) of all organs; all tissues from piglet 4 were positive for SVA and only 1 tissue sample from 3 piglets (nos. 2, 3, and 10) yielded negative results. Moreover, the nucleic acids of all other viruses investigated during this study were not amplified.

**Figure 2:**
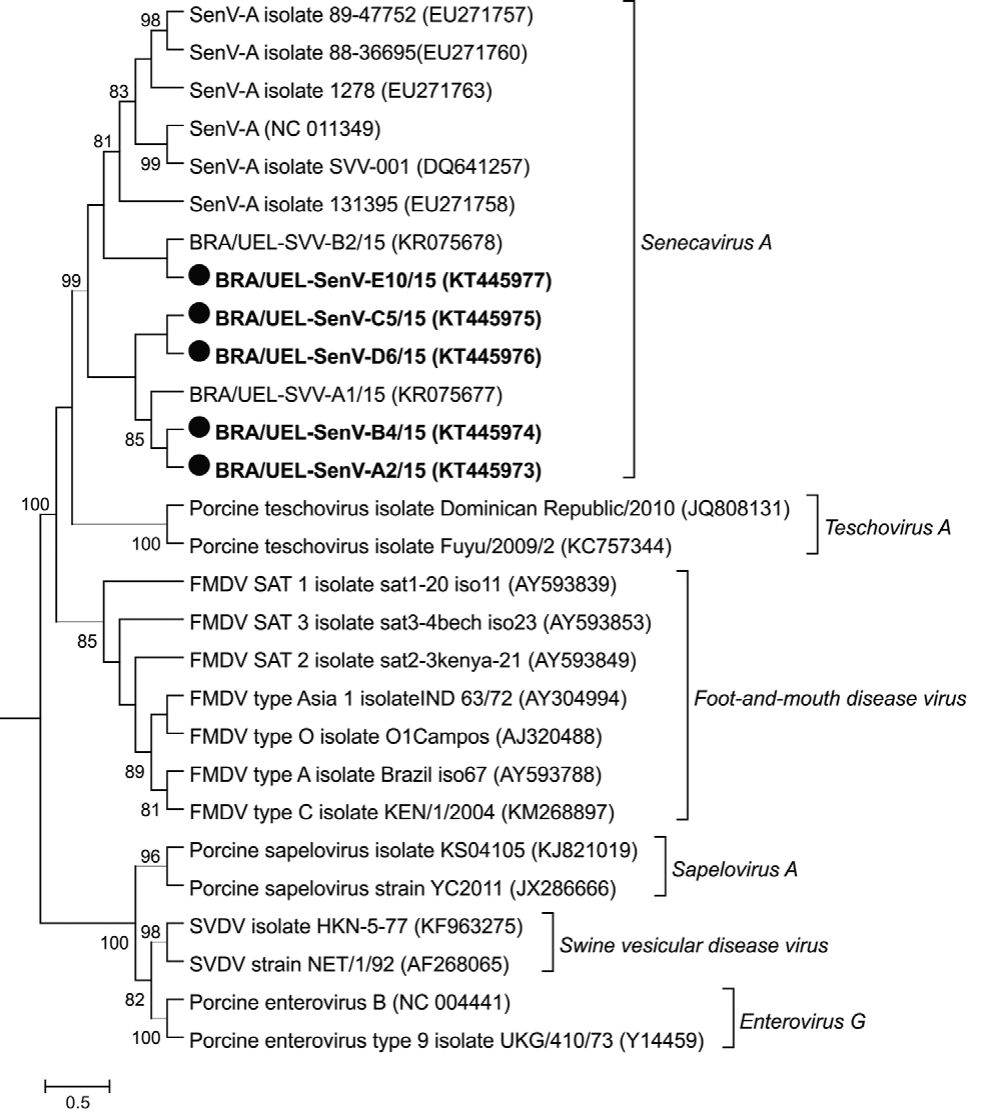
Phylogenetic relationship of strains of Senecavirus A identified in Brazil during 2015 (black circles) and other sequences available in GenBank derived from species of picornavirus associated with vesicular disease. Maximum-likelihood phylogenetic tree construction used the Kimura 2-parameter model with γ distribution based on the partial viral protein (VP) 3/VP1 region of the Senecavirus A genome. GenBank accession numbers are given in parentheses. Bootstrap values determined in 1,000 replication. Scale bar indicates nucleotide substitutions per site.

Sequence analysis from the 17 amplicons showed 98.8%–100% nt and aa similarities between each other and other isolates from Brazil available in GenBank (accession nos. KR075677 and KR075678). The SVA isolates we identified had similarities that varied from 87.4% nt (GenBank accession no. EU271760) to 98.5% nt (GenBank accession no. KC667560) and 94.4% aa (GenBank accession nos. EU271759 and EU271760) to 99.4% aa (GenBank accession no. KC667560) for isolates identified in North America. Phylogenetic analysis showed that the strains from this study (GenBank accession no. KT445973–KT445977) clustered with other known isolates of SVA and were distant from other picornaviruses associated with vesicular diseases (Figure 2).

## Conclusions

SVA has been associated with PIVD in pigs with vesicular lesions at the snout, coronary band, and hooves (*1*–*3*). However, findings from our investigation suggest a new clinical syndrome associated with SVA infection that resulted in disease to multiple tissues and organs of these piglets.

The patterns of the cutaneous lesions identified in this study might be similar to those of other vesicular infections of picornavirus (FMDV and SVDV), in which ballooning degeneration of epithelial cells and the formation of microvesicles are hallmarks (*14*,*15*). In addition, FMDV and SVDV affect different organs of susceptible animals—the heart, lungs, lymph nodes, bone marrow, and central nervous system (*14*,*15*)—suggesting a wide organ tropism of these viruses.

An interesting feature during this study was the constant immunolabelling of SVA within epithelial cells of the choroid plexus of the brain and the surrounding endothelia of blood vessels in piglets with neurologic disease. On the basis of the IHC results and molecular findings in different tissues of the brain, we theorized that the neurologic manifestations of SVA observed during this investigation might be due to early infection of the choroid plexus through alteration of the integrity of the vascular epithelium and subsequent dissemination to the adjacent neuropil. The IHC detection of SVA within the urinary epithelium of all piglets suggests that urine might be a mode of dissemination and a possible source of contamination within affected pig farms.

Another unusual finding associated with SVA infection during this study was the occurrence of diarrhea in piglets. Molecular screening did not detect any of the common enteric viral pathogens of suckling piglets. However, the IHC and reverse transcription PCR identified SVA in the small intestine of piglets with diarrhea, demonstrating the ability of SVA to replicate within the enteric epithelium.

Our results suggest that SVA is a pantropic virus that produces a multisystemic disease entity in pigs infected at an early age. The constant immunolabelling of the uroepithelium of all piglets with SVA antigens might indicate that in-pen contamination, through urine, should be considered as a possible route for the dissemination of this virus.
